# Comparing the burdens of opportunistic infections among patients with systemic rheumatic diseases: a nationally representative cohort study

**DOI:** 10.1186/s13075-019-1997-5

**Published:** 2019-10-12

**Authors:** Chung-Yuan Hsu, Chi-Hua Ko, Jiun-Ling Wang, Tsai-Ching Hsu, Chun-Yu Lin

**Affiliations:** 1 Division of Rheumatology, Allergy, and Immunology, Department of Internal Medicine, Kaohsiung Chang Gung Memorial Hospital and Chang Gung University College of Medicine, Kaohsiung, Taiwan; 2 Department of Life Sciences, National Chung Hsing University, Taichung, Taiwan; 3 Department of Rheumatology, Allergy and Immunology, Chang Gung Memorial Hospital, Yunlin, Taiwan; 4 Department of Internal Medicine, National Cheng Kung University Hospital, College of Medicine, National Cheng Kung University, No.138, Sheng Li Road, 704 Tainan, Taiwan; 5 Institute of Biochemistry, Microbiology and Immunology, Chung Shan Medical University, Taichung, Taiwan

**Keywords:** Polymyositis, Dermatomyositis, Systemic lupus erythematosus, Systemic rheumatic disease, Opportunistic infection

## Abstract

**Objective:**

To estimate and compare the burdens of opportunistic infections and herpes zoster in real-world practice among patients with various systemic rheumatic diseases.

**Methods:**

This 13-year cohort study used national health insurance data to compare the incidence rates (IRs) of nine opportunistic infections among patients with five rheumatic diseases. The analyses were stratified according to follow-up duration using Poisson regression, and Cox models were used to compare the risk of first opportunistic infection.

**Results:**

During 2000–2013, we identified 76,966 patients who had polymyositis/dermatomyositis (PM/DM, 2270 cases), systemic lupus erythematosus (SLE, 15,961 cases), systemic sclerosis (SSc, 2071 cases), rheumatoid arthritis (RA, 38,355 cases), or primary Sjögren’s syndrome (pSS, 18,309 cases). The IR of opportunistic infections was highest for PM/DM cases (61.3/1000 person-years, 95% confidence interval [CI] 56.6–66.2), followed by SLE cases (43.1/1000 person-years, 95% CI 41.7–44.5), SSc cases (31.6/1000 person-years, 95% CI 28.3–35.1), RA cases (25.0/1000 person-years, 95% CI 24.4–25.7), and pSS cases (24.1/1000 person-years, 95% CI 23.1–25.2). Multivariable Cox analysis revealed that, relative to SLE, PM/DM was associated with a significantly higher risk of opportunistic infections (hazard ratio 1.18, 95% CI 1.08–1.29). The risk of opportunistic infections was highest during the first year after the diagnosis of all five rheumatic diseases.

**Conclusions:**

The risk of opportunistic infection was highest for PM/DM, followed by SLE, SSc, RA, and pSS. Careful observation and preventive therapy for opportunistic infections may be warranted in selected PM/DM patients, especially during the first year after the diagnosis.

## Introduction

Infection remains a leading cause of morbidity, hospitalization, and mortality among patients with autoimmune rheumatic disease [1, 2]. Furthermore, the estimated rates of infectious complications can be 26–50% among patients with polymyositis/dermatomyositis (PM/DM) or systemic lupus erythematosus (SLE) [3–5]. Several factors may influence the vulnerability of patients with rheumatic diseases, with infectious diseases being strongly associated with their frequent use of corticosteroids and immunosuppressive agents. There is also evidence that innate and adaptive immunity against various pathogens is impaired in patients with SLE [6]. Moreover, the development of infection in patients with rheumatic diseases leads to a much poorer prognosis relative to that of patients without infectious diseases [2, 7].

In addition to common infections, opportunistic infection (OI) has emerged as an important complication in developed countries [8]. Interestingly, the risks of herpes zoster and *Pneumocystis jiroveci* pneumonia are elevated among SLE patients [9, 10], while DM was recently shown to be associated with elevated rates of herpes zoster and tuberculosis [11, 12]. However, most previous studies regarding the relationship between rheumatic diseases and OI were focused on SLE and were limited by their small-scale or single-centre designs. Furthermore, there is scarce research regarding the incidence rates (IRs) of OI in other major connective tissue diseases, such as systemic sclerosis (SSc) and primary Sjögren’s syndrome (pSS). Moreover, among patients with PM/DM or SLE, there are no large-scale studies regarding the incidences of other OIs (e.g. aspergillosis, cryptococcosis, non-tuberculous mycobacteria, and cytomegalovirus infection). We are also unaware of any studies regarding whether the burden of OI varies among different connective tissue diseases. Therefore, the present study aimed to determine the incidence rates of various OIs in real-world practice among Taiwanese patients with five systemic rheumatic diseases (SLE, PM/DM, SSc, pSS, and rheumatoid arthritis [RA]). We also compared the risks of OIs between these rheumatic diseases.

## Methods

### Data source

This retrospective cohort study evaluated data from Taiwan’s National Health Insurance Research Database (NHIRD; http://nhird.nhri.org.tw/en/index.html), which is maintained by Taiwan’s National Health Research Institutes (NHRI). The NHIRD contains detailed demographic and healthcare information, including relevant diagnostic and procedural codes, for > 23,000,000 individuals (approximately 99% of Taiwan’s population). Furthermore, the size of the NHIRD has led to its extensive use for epidemiological studies [13], which have validated its accuracy for identifying major diseases, such as diabetes mellitus and cerebrovascular disease [14, 15]. The study’s retrospective protocol to evaluate de-identified secondary data was approved by the institutional review board of National Cheng Kung University Hospital (B-EX-108-012).

### Patients

The five rheumatic diseases were identified using codes from the International Classification of Diseases, ninth revision, clinical modification (ICD-9-CM). Thus, inpatient and outpatient care claims were searched to identify cases involving SLE (710.0), RA (714.0), SSc (710.1), pSS (710.2), and PM/DM (710.4 and 710.3). To increase the specificity of case ascertainment for the five rheumatic diseases, we also evaluated catastrophic illness certificates, which are issued by the Bureau of National Health Insurance to patients with autoimmune diseases (e.g. SLE, RA, PM/DM, pSS, or SSc) in order to exempt them from co-payment requirements for related medical care. The certificate is only issued to the patient when their medical records, laboratory data, and imaging results have been reviewed by two independent rheumatologists, who confirm that the corresponding classification criteria have been fulfilled. For example, the certificate can be issued to SLE patients when their symptoms, laboratory findings, and radiographic findings fulfill the 1997 American College of Rheumatology Revised Criteria for Classification of Systemic Lupus Erythematosus [16]. In RA cases, the American Rheumatism Association 1987 revised criteria or the 2010 American College of Rheumatology/European League Against Rheumatism criteria must be fulfilled [17, 18]. The revised American–European Consensus Group Classification Criteria or the European classification criteria are used for pSS [19, 20], the 1980 systemic sclerosis classification criteria are used for SSc [21], and Bohan and Peter’s criteria are used for PM/DM [22, 23]. Patients without catastrophic illness certificates were excluded from our study.

The present study only included incident cases of autoimmune rheumatic diseases and excluded patients with ≥ 2 rheumatic diagnoses to ensure that we did not consider patients with secondary Sjögren’s syndrome or overlapping syndromes. The index date was defined as the first diagnosis of autoimmune disease between January 1, 2000, and December 31, 2013. Patients were followed up until the last episode of OI, death, or the end date (December 31, 2013).

### Identification of OI

Based on the ICD-9-CM codes and the 2015 consensus recommendations for infection reporting [8], the OI types were defined as candidiasis (112), aspergillosis (117.3, 484.6), *Cryptococcus* infection (117.5, 321.0), *Pneumocystis jiroveci* pneumonia (136.3), cytomegalovirus infection (078.5, 771.1), salmonellosis (003), tuberculosis (010–018), non-tuberculous *Mycobacterium* infection (031.0, 031.2, 031.8, 031.9), herpes zoster (053), toxoplasmosis (130), coccidioidomycosis (114), and histoplasmosis (115). These codes for OI were generally considered to be accurate in the administrative database [24, 25], although case ascertainment was improved by restricting the eligible cases to those that involved inpatient claims using these codes, with the exception of herpes zoster, which generally does not require hospitalization. Patients were allowed to have multiple OI types or multiple episodes of the same OI, although only the first episode for each OI type was considered in the analysis of cases with multiple episodes of the same OI. Patients who had experienced OI before the index date were excluded from the analysis.

### Covariate information

Patient characteristics (age, sex, income level, and comorbidities) were retrieved, and income level was used as a surrogate for socioeconomic status by categorizing the average monthly income as low (≤ 19,200 New Taiwan dollars [NTDs]), intermediate (19,201–40,000 NTD), and high (> 40,000 NTD). The selected baseline comorbidities were identified using ICD-9-CM codes for diabetes (250), chronic kidney disease (580–587), hypertension (401–405), ischaemic heart disease (410–414), cancer (140–208), dyslipidaemia (272), congestive heart failure (428), chronic obstructive lung disease (491, 492, 496), cerebrovascular disease (430–438), peripheral artery disease (443), liver cirrhosis (571.2, 571.5, 571.6), hepatitis B virus infection (070.2, 070.3, v02.61), hepatitis C virus infection (070.41, 070.44, 070.51, 070.54, 070.70, 070.71, v02.62), dementia (290, 294.1, 294.2, 331.0), and depression (296.2, 296.3, 311). Comorbidities were considered present if the corresponding code was used for a single inpatient claim or ≥ 3 outpatient visits. We also recorded the use of various medications within 90 days of the index date, including systemic corticosteroid, cyclophosphamide, methotrexate, azathioprine, cyclosporine, and leflunomide.

### Statistical analysis

Demographic data and baseline comorbidities were presented as mean ± standard deviation for continuous variables or as number (percentage) for categorical variables. These variables were then analysed using Student’s independent *t* test or Pearson’s chi-squared test. Incidence rates (IRs) for overall OIs, and individual OI types, were estimated by dividing the total number of OI episodes by the relevant person-years value during the observation period. The time from rheumatic disease diagnosis to OI occurrence was stratified as 0–1 year, 1–2 years, 2–3 years, 4–5 years, and > 5 years, and the incidence rate ratios (IRRs) for OI at the various follow-up times were estimated using Poisson regression. The Kaplan-Meier method and log-rank test were used to identify differences in the cumulative incidences of OI between the five rheumatic diseases. Cox proportional hazard regression analyses were performed to estimate the effects of each disease on the risk of the first OI episode, after adjusting for age, sex, income level, and comorbidities. Crude and adjusted hazard ratios (HRs and aHRs) with 95% confidence intervals (CIs) were used to describe the magnitudes of these effects.

### Sensitivity analysis

To investigate the robustness of the main findings, sensitivity analyses were designed by excluding herpes zoster from the definition of OI. The same methods were then used to determine estimates of OI risk among patients with the various rheumatic diseases. An additional sensitivity analysis was performed to generate subdistribution hazard ratios (sHRs) via the competing risk regression model using Fine and Gray’s method [26], with death as the competing risk. A two-sided *P* value of < 0.05 was considered significant. All data management and statistical analyses were performed using Stata 13 software (StataCorp, College Station, TX, USA).

## Results

### Patient characteristics

Between January 1, 2000, and December 31, 2013, we identified 76,966 patients with rheumatic diseases, including 15,961 SLE cases, 38,355 RA cases, 18,309 pSS cases, 2071 SSc cases, and 2270 PM/DM cases. Table 1 shows the patients’ demographic characteristics, comorbidities, medications, and mean follow-up duration. Female sex was the predominant factor for all five rheumatic diseases. The highest female-to-male ratios were observed for SLE and pSS. The mean age was lowest in SLE cases (37.2 ± 17.0 years) and was highest in pSS cases (54.7 ± 14.3 years). Diabetes, hypertension, and dyslipidaemia were the most common comorbidities, and the proportion of patients with cancer was highest among patients with PM/DM, while that of patients with depression was highest among patients with pSS. Patients with RA, SLE, and SSc had longer follow-up times than those with PM/DM and pSS. Additional file 1: Table S1 shows the calendar year distributions of the index dates for patients with each rheumatic disease.

**Table 1:** Patient characteristics according to systemic rheumatic disease

Variable	SLE (*N* = 15,961)	PM/DM (*N* = 2270)	SSc (*N* = 2071)	RA (*N* = 38,355)	pSS (*N* = 18,309)
Sex, *n* (%)					
Male	2027 (12.7)	760 (33.5)	544 (26.3)	9013 (23.5)	2032 (11.1)
Female	13,934 (87.3)	1510 (66.5)	1527 (73.7)	29,342 (76.5)	16,277 (88.9)
Age in years, mean ± SD	37.2 ± 17.0	47.8 ± 17.7	51.8 ± 15.3	53.3 ± 15.4	54.7 ± 14.3
Age group, *n* (%)					
0–16 years	1342 (8.4)	148 (6.5)	32 (1.6)	712 (1.8)	42 (0.2)
16–45 years	9853 (61.7)	767 (33.8)	610 (29.4)	9688 (25.3)	4363 (23.8)
45–65 years	3512 (22.1)	975 (43.0)	1000 (48.3)	19,105 (49.8)	9456 (51.6)
> 65 years	1254 (7.8)	380 (16.7)	429 (20.7)	8850 (23.1)	4448 (24.4)
Income in NTD, *n* (%)					
Low (< 19,200)	8092 (50.7)	1270 (56.0)	1129 (54.5)	20,647 (53.8)	9521 (52.0)
Intermediate (19,201–40,000)	6071 (38.1)	747 (32.9)	721 (34.8)	14,024 (36.5)	6526 (35.6)
High (> 40,000)	1798 (11.2)	253 (11.1)	221 (10.7)	3684 (9.7)	2262 (12.4)
Comorbidities, *n* (%)					
Diabetes mellitus	807 (5.1)	255 (11.3)	226 (11.0)	4528 (11.8)	2116 (11.5)
Hypertension	2574 (16.1)	536 (23.6)	556 (26.8)	10,485 (27.3)	5277 (28.8)
Chronic kidney disease	1390 (8.7)	99 (4.4)	147 (7.1)	1839 (4.8)	1064 (5.8)
COPD	862 (5.4)	240 (10.6)	273 (13.2)	3930 (10.3)	2218 (12.1)
Ischaemic heart disease	971 (6.1)	264 (11.6)	282 (13.6)	4643 (12.1)	2892 (15.9)
Dyslipidaemia	1202 (7.5)	381 (16.8)	315 (15.3)	5743 (14.9)	3634 (19.8)
CHF	541 (3.4)	105 (4.6)	147 (7.1)	1303 (3.4)	584 (3.2)
Liver cirrhosis	270 (1.69)	37 (1.6)	58 (2.8)	444 (1.1)	401 (2.2)
Cerebrovascular disease	7.3 (4.4)	101 (4.5)	126 (6.1)	2235 (5.8)	1658 (9.1)
Cancer	739 (4.6)	279 (12.3)	128 (6.2)	2116 (5.5)	1567 (8.5)
Peripheral artery disease	942 (5.9)	140 (6.2)	624 (30.1)	1314 (3.4)	1145 (6.25)
HBV infection	317 (2.0)	113 (4.9)	46 (2.2)	1118 (2.9)	727 (3.9)
HCV infection	249 (1.6)	54 (2.4)	39 (1.9)	877 (2.3)	705 (3.8)
Depression	735 (4.6)	109 (4.8)	110 (5.3)	2265 (5.9)	2262 (12.3)
Dementia	92 (0.6)	13 (0.6)	12 (0.6)	322 (0.8)	279 (1.5)
Medications, *n* (%)					
Corticosteroid	13,846 (86.7)	2146 (94.5)	1442 (69.6)	29,284 (76.3)	8912 (48.7)
Cyclophosphamide	1320 (8.3)	208 (9.2)	186 (9.0)	192 (0.5)	229 (1.25)
Methotrexate	626 (3.9)	668 (29.4)	155 (7.5)	21,310 (55.6)	829 (4.5)
Azathioprine	3965 (24.8)	708 (31.2)	166 (8.0)	621 (1.6)	1104 (6.0)
Cyclosporine	270 (1.7)	99 (4.4)	37 (1.8)	1105 (2.9)	104 (0.57)
Leflunomide	35 (0.21)	15 (0.7)	11 (0.5)	1914 (5.0)	108 (0.59)
Length of follow-up (years), mean ± SD	5.6 ± 4.2	4.5 ± 4.1	5.3 ± 4.0	5.9 ± 3.9	4.5 ± 3.5

*SLE* systemic lupus erythematosus, *PM/DM* polymyositis/dermatomyositis, *RA* rheumatoid arthritis, *pSS* primary Sjögren’s syndrome, *SSc* systemic sclerosis, *SD* standard deviation, *NTD* New Taiwan dollars, *COPD* chronic obstructive pulmonary disease, *CHF* congestive heart failure, *HBV* hepatitis B virus, *HCV* hepatitis C virus

### Incidence of OI

We identified 13,002 episodes in 11,554 patients with OI. Among these patients, 10,341 cases (89.5%) involved 1 episode, 1020 cases (8.8%) involved 2 episodes, and 193 cases (1.7%) involved ≥ 3 episodes. Table 2 shows the IR and 95% CI values for total OI and the various OI types according to the patients’ rheumatic diseases, although < 2 events were detected for toxoplasmosis, coccidioidomycosis, and histoplasmosis, which were omitted from the analyses. The highest IR for total OI was observed in PM/DM cases (61.3/1000 person-years, 95% CI 56.6–66.2), which were followed by SLE cases (43.1/1000 person-years, 95% CI 41.7–44.5), SSc cases (31.6/1000 person-years, 95% CI 28.3–35.1), RA cases (25.0/1000 person-years, 95% CI 24.4–25.7), and pSS cases (24.1/1000 person-years, 95% CI 23.1–25.2). Relative to the SLE cohort, the IRR for OI in the PM/DM cohort was 1.42 (95% CI 1.31–1.55) (Table 3). Sub-analyses of the OI types revealed generally similar rankings from highest to lowest (PM/DM followed by SLE, SSc, RA, and pSS), with the exception that the IR for salmonellosis was highest in the SLE cohort.

**Table 2:** Incidence rates of opportunistic infections according to systemic rheumatic disease

	SLE (*N* = 15,961, PY = 89,256)		PM/DM (*N* = 2270, PY = 10,252)		SSc (*N* = 2071, PY = 10,868)		RA (*N* = 38,355, PY = 22,7549)		pSS (*N* = 18,309, PY = 81,995)	
	Events	IR (95% CI)	Events	IR (95% CI)	Events	IR (95% CI)	Events	IR (95% CI)	Events	IR (95% CI)
Fungus										
Aspergillus	16	0.18 (0.10–0.29)	6	0.59 (0.22–1.27)	1	0.09 (0.01–0.51)	22	0.10 (0.06–0.15)	5	0.06 (0.02–0.14)
Candidiasis	388	4.35 (3.93–4.80)	85	8.29 (6.62–10.25)	49	4.51 (3.34–5.96)	426	1.87 (1.70–2.06)	145	1.77 (1.49–2.08)
Cryptococcus	39	0.44 (0.31–0.60)	9	0.88 (0.40–1.67)	2	0.18 (0.02–0.67)	45	0.20 (0.14–0.27)	14	0.17 (0.09–0.29)
PJP	45	0.51 (0.37–0.67)	18	1.76 (1.04–2.78)	4	0.37 (0.10–0.94)	18	0.08 (0.05–0.13)	9	0.11 (0.05–0.21)
Subtotal	488	5.48 (4.99–5.98)	118	11.5 (9.53–13.8)	56	5.15 (3.89–6.69)	511	2.25 (2.06–2.45)	173	2.11 (1.81–2.45)
CMV	100	1.12 (0.91–1.37)	18	1.76 (1.04–2.78)	4	0.37 (0.10–0.94)	29	0.13 (0.09–0.18)	15	0.18 (0.10–0.30)
Salmonellosis	272	3.05 (2.70–3.43)	30	2.93 (1.97–4.18)	11	1.01 (0.51–1.81)	118	0.52 (0.43–0.62)	27	0.33 (0.22–0.48)
Herpes zoster	2580	28.9 (27.8–30.0)	377	36.8 (33.2–40.7)	216	19.9 (17.3–22.7)	4084	17.9 (17.4–18.5)	1502	18.3 (17.4–19.3)
TB	341	3.82 (3.43–4.25)	71	6.93 (5.41–8.74)	47	4.32 (3.18–5.75)	839	3.69 (3.44–3.95)	213	2.60 (2.26–2.97)
NTM	63	0.71 (0.54–0.90)	14	1.37 (0.75–2.29)	9	0.82 (0.38–1.57)	121	0.53 (0.44–0.64)	48	0.59 (0.43–0.78)
Non-herpes zoster infection	1264	14.2 (13.4–15.0)	251	24.5 (21.6–27.7)	127	11.7 (9.74–13.9)	1617	7.10 (6.76–7.40)	476	5.81 (5.30–6.35)
Total	3844	43.1 (41.7–44.5)	628	61.3 (56.6–66.2)	343	31.6 (28.3–35.1)	5701	25.0 (24.4–25.7)	1978	24.1 (23.1–25.2)

Incidence rates (IRs) are reported as the number of cases per 1000 person-years

*SLE* systemic lupus erythematosus, *RA* rheumatoid arthritis, *pSS* primary Sjögren’s syndrome, *SSc* systemic sclerosis, *PM/DM* polymyositis/dermatomyositis, *PJP Pneumocystis jiroveci* pneumonia, *CMV* cytomegalovirus, *TB* tuberculosis, *NTM* non-tuberculosis mycobacterium, *PY* person-years

**Table 3:** Unadjusted incidence rate ratio for total and non-herpes zoster opportunistic infections in patients with different systemic rheumatic diseases

	Incidence rate ratio	95% CI	*P* value
Total opportunistic infections			
SLE	1 (reference)	–	–
PM/DM	1.42	1.31–1.55	< 0.001
SSc	0.73	0.65–0.85	< 0.001
RA	0.58	0.56–0.61	< 0.001
pSS	0.56	0.53–0.59	< 0.001
Non-herpes zoster opportunistic infections			
SLE	1 (reference)	–	–
PM/DM	1.73	1.50–1.98	< 0.001
SSc	0.83	0.68–0.99	0.035
RA	0.50	0.46–0.54	< 0.001
pSS	0.41	0.37–0.45	< 0.001

*SLE* systemic lupus erythematosus, *RA* rheumatoid arthritis, *pSS* primary Sjögren’s syndrome, *SSc* systemic sclerosis, *PM/DM* polymyositis/dermatomyositis, *CI* confidence interval

Table 4 shows the IRRs for OI according to the follow-up period. The risk of OI was highest during the first year after the diagnosis of the rheumatic diseases, with gradually decreasing risk over time. The lowest IR values for OI were observed at > 5 years after the diagnosis of PM/DM, SLE, RA, and pSS.

**Table 4:** Incidence rates and ratios for total opportunistic infections and non-herpes zoster infections according to systemic rheumatic disease and follow-up duration

Follow-up years	Person-year	Total opportunistic infections			Non-herpes zoster opportunistic infections		
		Events	IR	IRR	Events	IR	IRR
SLE							
0–1	14,355	1212	84.4	2.76 (2.53–2.99)	425	29.6	2.86 (2.48–3.31)
1–2	12,495	540	43.2	1.41 (1.27–1.57)	168	13.5	1.30 (1.07–1.58)
2–3	11,050	418	37.8	1.24 (1.10–1.38)	126	11.4	1.10 (0.89–1.36)
3–4	9729	359	36.9	1.20 (1.06–1.36)	106	10.9	1.05 (0.84–1.31)
4–5	8483	300	35.3	1.15 (1.01–1.31)	96	11.3	1.09 (0.86–1.38)
> 5	33,144	1015	30.6	1 (reference)	343	10.4	1 (reference)
Total	89,256	3844	43.1	–	1264	14.2	–
RA							
0–1	36,271	1123	30.9	1.32 (1.23–1.43)	331	9.13	1.44 (1.25–1.66)
1–2	32,300	825	25.5	1.09 (1.01–1.18)	226	7.00	1.10 (0.94–1.29)
2–3	28,646	676	23.6	1.01 (0.92–1.10)	201	7.02	1.11 (0.94–1.31)
3–4	25,375	596	23.5	1.01 (0.91–1.10)	165	6.51	1.03 (0.86–1.22)
4–5	22,283	547	24.5	1.05 (0.95–1.15)	170	7.63	1.20 (1.01–1.43)
> 5	82,674	1934	23.4	1 (reference)	524	6.34	1 (reference)
Total	227,549	5701	25.0	–	1617	7.10	–
pSS							
0–1	16,658	528	31.7	1.54 (1.36–1.75)	152	9.12	1.85 (1.44–2.37)
1–2	13,798	355	25.7	1.25 (1.08–1.43)	71	5.15	1.04 (0.76–1.41)
2–3	11,438	242	21.1	1.03 (0.88–1.20)	49	4.28	0.87 (0.61–1.22)
3–4	9386	196	20.8	1.01 (0.85–1.20)	47	5.01	1.01 (0.71–1.43)
4–5	7657	182	23.7	1.15 (0.97–1.37)	43	5.62	1.14 (0.78–1.62)
> 5	23,058	475	20.6	1 (reference)	114	4.94	1 (reference)
Total	81,995	1978	24.1	–	476	5.81	–
SSc							
0–1	1875	80	42.6	1.61 (1.18–2.18)	34	18.1	1.68 (1.04–2.72)
1–2	1600	57	35.6	1.34 (0.95–1.88)	18	11.2	1.04 (0.56–1.86)
2–3	1380	46	33.3	1.26 (0.87–1.80)	16	11.5	1.08 (0.56–1.96)
3–4	1183	40	33.8	1.27 (0.86–1.85)	11	9.29	0.86 (0.40–1.71)
4–5	1024	19	18.5	0.70 (0.40–1.15)	7	6.83	0.63 (0.24–1.43)
> 5	3806	101	26.5	1 (reference)	41	10.7	1 (reference)
Total	10,868	343	31.6	–	127	11.7	–
PM/DM							
0–1	1882	277	147.2	5.47 (4.31–6.98)	137	72.8	11.2 (7.14–18.2)
1–2	1510	100	66.2	2.46 (1.84–3.29)	36	23.8	3.66 (2.11–6.46)
2–3	1277	78	61.1	2.27 (1.66–3.09)	23	18.8	2.76 (1.48–5.15)
3–4	1098	42	38.2	1.42 (0.96–2.06)	17	15.4	2.37 (1.19–4.64)
4–5	957	36	37.6	1.40 (0.92–2.07)	15	15.6	2.40 (1.17–4.81)
> 5	3528	95	26.9	1 (reference)	23	6.51	1 (reference)
Total	10,252	628	61.3	–	127	24.5	–

IRs are reported as the number of cases per 1000 person-years

*IR* incidence rate, *IRR* incidence rate ratio, *SLE* systemic lupus erythematosus, *RA* rheumatoid arthritis, *pSS* primary Sjögren’s syndrome, *SSc* systemic sclerosis, *PM/DM* polymyositis/dermatomyositis

### Cumulative incidence and relative hazard of the first OI event

**Fig. 1:**
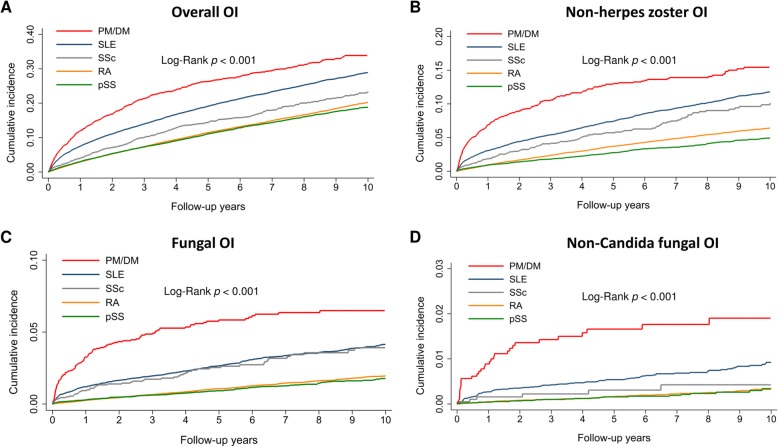
Comparing the cumulative incidences of **a** overall opportunistic infections (OI), **b** non-herpes zoster OIs, **c** fungal OIs, and **d** non-*Candida* fungal OIs. The patients were grouped according to their systemic rheumatic diseases: polymyositis/dermatomyositis (PM/DM), systemic lupus erythematosus (SLE), systemic sclerosis (SSc), rheumatoid arthritis (RA), and primary Sjögren’s syndrome (pSS)

Figure 1 shows the cumulative incidences of total OI over 10 years in the rheumatic disease cohorts. The highest risk was observed in the PM/DM cohort, which was followed by the SLE cohort, the SSc cohort, the RA cohort, and the pSS cohort (log-rank *P* < 0.001). We also examined the cumulative incidences of non-herpes zoster OIs, fungal OIs, and non-*Candida* fungal infections, which revealed similar risk patterns (Fig. 1b–d).

The Cox regression model, which was adjusted for age, sex, income level, and comorbidities, revealed that the risk of the first OI event was 18% higher in the PM/DM group than in the SLE group (aHR 1.18, 95% CI 1.08–1.29). Relative to the SLE group, reduced risks of OI were observed in the SSc group (aHR 0.58, 95% CI 0.51–0.65), the RA group (aHR 0.46, 95% CI 0.44–0.48), and the pSS group (aHR 0.42, 95% CI 0.39–0.44). Similar results were observed in the sensitivity analyses when herpes zoster was excluded from the outcome measurement (Table 5). The sensitivity analysis using the multivariable competing risk regression model also revealed that the PM/DM cohort had a significantly higher risk of OI than the SLE cohort (sHR 1.17, 95% CI 1.07–1.29), while lower risks of OI were observed in the SSc cohort (sHR 0.67, 95% CI 0.59–0.75), the RA cohort (sHR 0.59, 95% CI 0.57–0.62), and the pSS cohort (sHR 0.56, 95% CI 0.53–0.59).

**Table 5:** Comparing the risks of opportunistic infection according to systemic rheumatic disease by Cox regression and competing risk regression models

	Cox regression		Competing risk regression	
	Crude HR	Adjusted HR	Crude sHR	Adjusted sHR
Opportunistic infections				
SLE	1 (reference)	1 (reference)	1 (reference)	1 (reference)
PM/DM	1.38 (1.26–1.51)	1.18 (1.08–1.29)	1.22 (1.11–1.34)	1.17 (1.07–1.29)
SSc	0.75 (0.67–0.85)	0.58 (0.51–0.65)	0.70 (0.62–0.78)	0.67 (0.59–0.75)
RA	0.61 (1.26–1.51)	0.46 (0.44–0.48)	0.62 (0.59–0.65)	0.59 (0.57–0.62)
pSS	0.57 (0.54–0.60)	0.42 (0.39–0.44)	0.58 (0.55–0.61)	0.56 (0.53–0.59)
Non-herpes zoster opportunistic infections				
SLE	1 (reference)	1 (reference)	1 (reference)	1 (reference)
PM/DM	1.63 (1.41–1.89)	1.22 (1.05–1.42)	1.44 (1.24–1.68)	1.22 (1.05–1.43)
SSc	0.83 (0.68–1.01)	0.55 (0.45–0.67)	0.77 (0.64–0.93)	0.65 (0.54–0.79)
RA	0.50 (0.47–0.54)	0.33 (0.30–0.36)	0.51 (0.47–0.55)	0.45 (0.42–0.49)
pSS	0.38 (0.34–0.42)	0.25 (0.22–0.28)	0.39 (0.34–0.43)	0.35 (0.31–0.39)

*HR* hazard ratio, *sHR* subdistribution hazard ratio, *SLE* systemic lupus erythematosus, *RA* rheumatoid arthritis, *pSS* primary Sjögren’s syndrome, *SSc* systemic sclerosis, *PM/DM* polymyositis/dermatomyositis

## Discussion

To the best of our knowledge, this is the first nationally representative study to investigate the incidence of various OI types, including invasive fungal infection, mycobacterium infection, salmonellosis, and cytomegalovirus infection, among patients with five major immune-mediated diseases (PM/DM, SLE, RA, SSc, and pSS). Our study is also the first to demonstrate that the risks of OI vary for each specific disease, with the highest risk observed for PM/DM, followed by SLE, SSc, RA, and pSS. In addition, we found that the risk of OI was highest during the first year after the diagnosis of rheumatic disease, with the risk subsequently decreasing at longer intervals after the diagnosis.

Previous studies have indicated that SLE patients have higher rates of bacterial infection or OI than the general population [1, 2], which has been attributed to various factors. For example, several immune abnormalities have been reported in SLE patients, including complement deficiency [27], complement receptor deficiency [9, 28], defective chemotaxis and phagocytosis [7], decreased production of interleukin-8 by polymorphonuclear leukocytes [29], and impaired activity of T-helper cells against viral antigens [30]. Furthermore, disease-related factors can also increase the risk of OI in patients with SLE, with their lupus activity index independently predicting the risk of hospitalization for infectious disease [9]. Frequent use of glucocorticoids and immunosuppressive agents is also an important risk factor for unusual infection, and it has been reported that cyclophosphamide use for serious SLE manifestations is linked to fatal OIs [31]. A recent observational study also revealed that corticosteroid use had a dose-dependent effect on the rate of OIs [10].

One of the present study’s main findings was that the IR of OI was significantly higher for PM/DM than for SLE, even after adjusting for age, sex, and comorbidities. There are several possible explanations for this result. First, interstitial lung disease is a serious complication in up to 40–65% of PM/DM cases [32, 33], and patients with interstitial lung disease may be vulnerable to pulmonary infections by *Mycobacterium* and *Aspergillus* species [34, 35]. Second, PM/DM patients often require more intensive immunosuppression than SLE patients, and fatal refractory interstitial lung disease associated with PM/DM is not uncommon, with Kameda et al. [36] reporting that treatment using cyclophosphamide plus glucocorticoids was only effective in 25% of these critical patients. Triple therapy using cyclophosphamide, cyclosporin A, and glucocorticoids has been suggested to increase the response rate in these refractory patients [37], although no combination treatments (e.g. > 2 immunosuppressive agents) have been suggested for SLE patients, even in cases with lupus nephritis [38]. Thus, intensive immunosuppression may expose PM/DM patients to a significantly higher risk of OI than SLE patients. Third, PM/DM are strongly associated with a broad range of malignancies [39], which could contribute to the increased risk of OI through the use of cytotoxic anti-cancer therapies. Interestingly, malignancy can be present at the onset of idiopathic inflammatory myositis or may develop before or after the diagnosis of PM/DM [39], although we found that the risk of OI remained higher for PM/DM patients than for SLE patients, even after adjusting our regression model for various cancer types. Fourth, involvement of the striated muscle at the oropharynx and upper third of the oesophagus can be observed in PM/DM patients, which can alter their ability to swallow and increase their risk of aspiration pneumonia [40]. Similarly, a small proportion of PM/DM patients experience thoracic muscle myopathy, which leads to ventilatory compromise, difficulty managing respiratory secretions, and an elevated risk of respiratory infection [3, 41].

Although infectious complications are more common in patients with connective tissue diseases, it is unclear whether the risk of OI varies over time. The present study revealed that the risk of OI was highest during the first year after the diagnosis of systemic rheumatic disease, especially among PM/DM patients, where the IR of OI during the first year after diagnosis was approximately 5.4 times greater than the IR at > 5 years after diagnosis. Similarly, a French study of 156 PM/DM patients revealed that 62.5% of the OI events occurred during the first year after the PM/DM diagnosis [42]. Another cohort study explored the risk of herpes virus infection in 134 DM patients and also indicated that the IR was highest during the first year after DM diagnosis [43]. Other research has evaluated the courses of adult and juvenile DM patients, and the results suggested that disease activity was highest during the 6–12 months after the DM diagnosis, with improvement apparently accompanied by corticosteroid treatment [44–46]. Moreover, the required dosage of corticosteroid immunosuppression for DM was lower after 12 months of use and remained relatively constant until 36 months of use [46]. However, some studies have indicated that 11–30% of PM/DM patients developed OI before starting immunosuppressive therapy [42, 47]. Therefore, both high-dose corticosteroid treatment and high disease activity may contribute to the enhanced risk of OI during the first year after PM/DM diagnosis.

The introduction of biological agents has been a major advance in the treatment of RA [48]. For example, tumour necrosis factor alpha (TNF-*α*) inhibitors have potent immunosuppressive effect in this setting and can prevent radiographic progression or induce clinical remission in RA patients [49]. However, infectious complications are important concerns when patients are receiving anti-TNF therapies [50], and there is evidence that anti-TNF therapies are associated with increased risks of serious infections that may require hospitalization [51–53]. In this context, etanercept and adalimumab were the first biologic agents approved for the treatment of severe RA in Taiwan and were widely used after 2004. However, we did not perform separate analyses of the risks of OI before and after the era of biological therapy in Taiwan, and caution should be exercised when interpreting our data regarding the incidence of OI in RA cases. It is important to note that RA patients receiving anti-TNF therapy or other biological agents may experience a higher risk of OI, relative to their apparent risk based on our findings.

Taiwan is a country with an intermediate burden of tuberculosis, based on an estimated IR of 68 cases per 100,000 population in 2011 [54]. Our study revealed that incidences of tuberculosis in all five rheumatic diseases were several times higher than that in the general population, with the highest risk observed in the PM/DM cohort. These findings agree with the results from previous record-linkage studies conducted in Western countries [55, 56].

The strength of the present study lies in the use of a nationally representative data source with long-term follow-up data, which allowed us to examine the risks of overall and specific OIs according to five systemic rheumatic diseases. Nevertheless, our findings must be interpreted in the light of several limitations. First, the dataset lacked information regarding the activity or severity of the rheumatic diseases. Second, we did not incorporate variables regarding the exposure of glucocorticoids and immunosuppressants into our regression model, which precluded an analysis of their influence on the risk of OI. However, given the time-varying nature of medication use, it would be more appropriate to adopt a case-control study design for assessing the impact of immunosuppressive drugs. Third, we only counted the first episode in cases with multiple episodes of the same OI, which suggests that our calculated IR values might be underestimated.

## Conclusion

This nationally representative cohort study revealed that patients with PM/DM had the highest risk of OI, followed by SLE, SSc, RA, and pSS, in order of decreasing risk. Furthermore, the highest risk of OI was observed during the first year after the diagnosis of systemic rheumatic disease, especially in cases of PM/DM. These findings highlight the importance of monitoring for OI development during the treatment of these autoimmune rheumatic diseases, especially for patients with PM/DM.

## Supplementary information

**Additional file 1: Table S1.** Case distributions according to the calendar year of the index date and the rheumatic diseases.

## Data Availability

The datasets used and/or analysed during the current study are available from the corresponding author on reasonable request.

## Abbreviations

CHF: Congestive heart failure
CMV: Cytomegalovirus
COPD: Chronic obstructive pulmonary disease
HBV: Hepatitis B virus
HCV: Hepatitis C virus
HR: Hazard ratios
ICD-9-CM: International Classification of Diseases, ninth revision, clinical modification
IR: Incidence rates
IRR: Incidence rate ratio
NHIRD: National Health Insurance Research Database
NHRI: National Health Research Institutes
NTD: New Taiwan dollars
NTM: Non-tuberculosis mycobacterium
OI: Opportunistic infection
PJP: *Pneumocystis jiroveci* pneumonia
PM/DM: Polymyositis/dermatomyositis
pSS: Primary Sjögren’s syndrome
PY: Person-years
RA: Rheumatoid arthritis
sHR: Subdistribution hazard ratio
SD: Standard deviation
SLE: Systemic lupus erythematosus
SSc: Systemic sclerosis
TB: Tuberculosis
TNF-*α*: Tumour necrosis factor alpha

## References

[1] Feldman CH, Hiraki LT, Winkelmayer WC (2015). Serious infections among adult Medicaid beneficiaries with systemic lupus erythematosus and lupus nephritis. Arthritis Rheumatol.

[2] Peng JM, Du B, Wang Q (2016). Dermatomyositis and polymyositis in the intensive care unit: a single-center retrospective cohort study of 102 patients. PLoS One.

[3] Marie I, Hatron PY, Dominique S (2001). Polymyositis and dermatomyositis: short term and long term outcome, and predictive factors of prognosis. J Rheumatol.

[4] Dankó K, Ponyi A, Constantin T (2004). Long-term survival of patients with idiopathic inflammatory myopathies according to clinical features: a longitudinal study of 162 cases. Medicine.

[5] Zandman-Goddard G, Shoenfeld Y (2005). Infections and SLE. Autoimmunity.

[6] Danza A, Ruiz-Irastorza G (2013). Infection risk in systemic lupus erythematosus patients: susceptibility factors and preventive strategies. Lupus.

[7] Petri M (1998). Infection in systemic lupus erythematosus. Rheum Dis Clin North Am.

[8] Winthrop KL, Novosad SA, Baddley JW (2015). Opportunistic infections and biologic therapies in immune-mediated inflammatory diseases: consensus recommendations for infection reporting during clinical trials and postmarketing surveillance. Ann Rheum Dis.

[9] Petri M, Genovese M (1992). Incidence of and risk factors for hospitalizations in systemic lupus erythematosus: a prospective study of the Hopkins lupus cohort. J Rheumatol.

[10] Yang SC, Lai YY, Huang MC, Tsai CS, Wang JL (2018). Corticosteroid dose and the risk of opportunistic infection in a national systemic lupus erythematosus cohort. Lupus.

[11] Tsai SY, Lin CL, Wong YC (2015). Increased risk of herpes zoster following dermatomyositis and polymyositis: a nationwide population-based cohort study. Medicine.

[12] Wu PH, Lin YT, Yang YH, Lin YC, Lin YC (2015). The increased risk of active tuberculosis disease in patients with dermatomyositis - a nationwide retrospective cohort study. Sci Rep.

[13] Chang SL, Huang YL, Lee MC (2018). Association of varicose veins with incident venous thromboembolism and peripheral artery disease. JAMA.

[14] Cheng CL, Kao YH, Lin SJ, Lee CH, Lai ML (2011). Validation of the national health insurance research database with ischemic stroke cases in Taiwan. Pharmacoepidemiol Drug Saf.

[15] Lin CC, Lai MS, Syu CY, Chang SC, Tseng FY (2005). Accuracy of diabetes diagnosis in health insurance claims data in Taiwan. J Formos Med Assoc.

[16] Hochberg MC (1997). Updating the American College of Rheumatology revised criteria for the classification of systemic lupus erythematosus. Arthritis Rheum.

[17] Arnett FC, Edworthy SM, Bloch DA (1988). The American Rheumatism Association 1987 revised criteria for the classification of rheumatoid arthritis. Arthritis Rheum.

[18] Aletaha D, Neogi T, Silman AJ (2010). 2010 Rheumatoid arthritis classification criteria: an American College of Rheumatology/European League Against Rheumatism collaborative initiative. Arthritis Rheum.

[19] Vitali C, Bombardieri S, Moutsopoulos HM (1993). Preliminary criteria for the classification of Sjögren’s syndrome. Results of a prospective concerted action supported by the European Community. Arthritis Rheum.

[20] Vitali C, Bombardieri S, Jonsson R (2002). Classification criteria for Sjögren’s syndrome: a revised version of the European criteria proposed by the American-European Consensus Group. Ann Rheum Dis.

[21] Preliminary criteria for the classification of systemic sclerosis (scleroderma) (1980). Subcommittee for scleroderma criteria of the American Rheumatism Association Diagnostic and Therapeutic Criteria Committee. Arthritis Rheum.

[22] Bohan A, Peter JB (1975). Polymyositis and dermatomyositis (first of two parts). N Engl J Med.

[23] Bohan A, Peter JB (1975). Polymyositis and dermatomyositis (second of two parts). N Engl J Med.

[24] Schneeweiss S, Robicsek A, Scranton R, Zuckerman D, Solomon DH (2007). Veteran's affairs hospital discharge databases coded serious bacterial infections accurately. J Clin Epidemiol.

[25] Grijalva CG, Chung CP, Stein CM (2008). Computerized definitions showed high positive predictive values for identifying hospitalizations for congestive heart failure and selected infections in Medicaid enrollees with rheumatoid arthritis. Pharmacoepidemiol Drug Saf.

[26] Fine JP, Gray RJ (1999). A proportional hazards model for the subdistribution of a competing risk. J Am Stat Assoc.

[27] Ross SC, Densen P (1984). Complement deficiency states and infection: epidemiology, pathogenesis and consequences of neisserial and other infections in an immune deficiency. Medicine.

[28] Wilson JG, Ratnoff WD, Schur PH, Fearon DT (1986). Decreased expression of the C3b/C4b receptor (CR1) and the C3d receptor (CR2) on B lymphocytes and of CR1 on neutrophils of patients with systemic lupus erythematosus. Arthritis Rheum.

[29] Hsieh SC, Tsai CY, Sun KH (1994). Decreased spontaneous and lipopolysaccharide stimulated production of interleukin 8 by polymorphonuclear neutrophils of patients with active systemic lupus erythematosus. Clin Exp Rheumatol.

[30] Bermas BL, Petri M, Goldman D (1994). T helper cell dysfunction in systemic lupus erythematosus (SLE): relation to disease activity. J Clin Immunol.

[31] Pryor BD, Bologna SG, Kahl LE (1996). Risk factors for serious infection during treatment with cyclophosphamide and high-dose corticosteroids for systemic lupus erythematosus. Arthritis Rheum.

[32] Fathi M, Dastmalchi M, Rasmussen E, Lundberg IE, Tornling G (2004). Interstitial lung disease, a common manifestation of newly diagnosed polymyositis and dermatomyositis. Ann Rheum Dis.

[33] Kang EH, Lee EB, Shin KC (2005). Interstitial lung disease in patients with polymyositis, dermatomyositis and amyopathic dermatomyositis. Rheumatology.

[34] Smith NL, Denning DW (2011). Underlying conditions in chronic pulmonary aspergillosis including simple aspergilloma. Eur Respir J.

[35] Park SW, Song JW, Shim TS (2012). Mycobacterial pulmonary infections in patients with idiopathic pulmonary fibrosis. J Korean Med Sci.

[36] Kameda H, Nagasawa H, Ogawa H (2005). Combination therapy with corticosteroids, cyclosporin A, and intravenous pulse cyclophosphamide for acute/subacute interstitial pneumonia in patients with dermatomyositis. J Rheumatol.

[37] Miyazaki E, Ando M, Muramatsu T (2007). Early assessment of rapidly progressive interstitial pneumonia associated with amyopathic dermatomyositis. Clin Rheumatol.

[38] Hahn BH, McMahon MA, Wilkinson A (2012). American College of Rheumatology guidelines for screening, treatment, and management of lupus nephritis. Arthritis Care Res.

[39] Sigurgeirsson B, Lindelöf B, Edhag O, Allander E (1992). Risk of cancer in patients with dermatomyositis or polymyositis. A population-based study. N Engl J Med.

[40] Mugii N, Hasegawa M, Matsushita T (2016). Oropharyngeal dysphagia in dermatomyositis: associations with clinical and laboratory features including autoantibodies. PLoS One.

[41] Wu Q, Wedderburn LR, McCann LJ (2017). Juvenile dermatomyositis: Latest advances. Best Pract Res Clin Rheumatol.

[42] Marie I, Hachulla E, Chérin P (2005). Opportunistic infections in polymyositis and dermatomyositis. Arthritis Rheum.

[43] Fardet L, Rybojad M, Gain M (2009). Incidence, risk factors, and severity of herpesvirus infections in a cohort of 121 patients with primary dermatomyositis and dermatomyositis associated with a malignant neoplasm. Arch Dermatol.

[44] Ramanan AV, Campbell-Webster N, Ota S (2005). The effectiveness of treating juvenile dermatomyositis with methotrexate and aggressively tapered corticosteroids. Arthritis Rheum.

[45] Schmeling H, Stephens S, Goia C (2011). Nailfold capillary density is importantly associated over time with muscle and skin disease activity in juvenile dermatomyositis. Rheumatology.

[46] Johnson NE, Arnold WD, Hebert D (2015). Disease course and therapeutic approach in dermatomyositis: a four-center retrospective study of 100 patients. Neuromuscul Disord.

[47] Viguier M, Fouéré S, de la Salmonière P (2003). Peripheral blood lymphocyte subset counts in patients with dermatomyositis: clinical correlations and changes following therapy. Medicine.

[48] Davignon JL, Rauwel B, Degboé Y (2018). Modulation of T-cell responses by anti-tumor necrosis factor treatments in rheumatoid arthritis: a review. Arthritis Res Ther.

[49] Stamm TA, Machold KP, Aletaha D (2018). Induction of sustained remission in early inflammatory arthritis with the combination of infliximab plus methotrexate: the DINORA trial. Arthritis Res Ther.

[50] Pawar A, Desai RJ, Solomon DH (2019). Risk of serious infections in tocilizumab versus other biologic drugs in patients with rheumatoid arthritis: a multidatabase cohort study. Ann Rheum Dis.

[51] Singh JA, Cameron C, Noorbaloochi S (2015). Risk of serious infection in biological treatment of patients with rheumatoid arthritis: a systematic review and meta-analysis. Lancet.

[52] Galloway JB, Hyrich KL, Mercer LK (2011). Anti-TNF therapy is associated with an increased risk of serious infections in patients with rheumatoid arthritis especially in the first 6 months of treatment: updated results from the British Society for Rheumatology Biologics Register with special emphasis on risks in the elderly. Rheumatology (Oxford).

[53] Smitten AL, Choi HK, Hochberg MC (2008). The risk of hospitalized infection in patients with rheumatoid arthritis. J Rheumatol.

[54] Taiwan Centers for Disease Control (2013). Taiwan Tuberculosis Control Report.

[55] Ramagopalan SV, Goldacre R, Skingsley A, Conlon C, Goldacre MJ (2013). Associations between selected immune-mediated diseases and tuberculosis: record-linkage studies. BMC Med.

[56] Airio A, Kauppi M, Kautiainen H, Hakala M, Kinnula V (2007). High association of mycobacterial infections with polymyositis in a non-endemic country for tuberculosis. Ann Rheum Dis.

