# Supplementary material for: Comparing the burdens of opportunistic infections among patients with systemic rheumatic diseases: a nationally representative cohort study
Source: Arthritis Res Ther. 2019 Oct 12;21:211. doi: 10.1186/s13075-019-1997-5 (PMC6790041; doi:10.1186/s13075-019-1997-5)
Supplement: Supplementary file 1 — Additional file 1: Table S1. Case distributions according to the calendar year of the index date and the rheumatic diseases. [file 13075_2019_1997_MOESM1_ESM.docx]

Additional file 1: Table S1. Case distributions according to the calendar year of the index date and the rheumatic diseases.

| Year of index date | SLE  (N=15,961) | PM/DM  (N=2270) | SSc  (N=2071) | RA  (N=38,355) | pSS  (N=18,309) |
| --- | --- | --- | --- | --- | --- |
| 2000-2003, n (%) | 5028 (31.5) | 664 (29.2) | 623 (30.1) | 11,121 (29.0) | 2608 (14.2) |
| 2004-2007, n (%) | 4455 (27.9) | 651 (28.7) | 586 (28.3) | 11,215 (29.2) | 4096 (22.4) |
| 2008-2010, n (%) | 3314 (20.8) | 483 (21.3) | 413 (19.9) | 8013 (20.9) | 4860 (26.6) |
| 2011-2013, n (%) | 3164 (19.8) | 472 (20.8) | 449 (21.7) | 8006 (20.9) | 6745 (36.8) |

SLE: systemic lupus erythematosus, PM/DM: polymyositis/dermatomyositis, SSc: systemic sclerosis, RA: rheumatoid arthritis, pSS: primary Sjögren’s syndrome.
